# Unveiling the Potential of Silymarin, *Spirulina platensis*, and *Chlorella vulgaris* towards Cardiotoxicity via Modulating Antioxidant Activity, Inflammation, and Apoptosis in Rats

**DOI:** 10.3390/life14101289

**Published:** 2024-10-11

**Authors:** Hanem F. El-Gendy, Hanem K. Khalifa, Ahmed Omran, Reda M. S. Korany, Shaimaa Selim, Eman Hussein, Rashed A. Alhotan, Anam Ayyoub, Shimaa R. Masoud

**Affiliations:** 1 Department of Pharmacology, Faculty of Veterinary Medicine, University of Sadat City, Sadat City 32897, Egypt; hanem.elgendy@vet.usc.edu.eg; 2 Department of Biochemistry and Chemistry of Nutrition, Faculty of Veterinary Medicine, University of Sadat City, Sadat City 32897, Egypt; hanem.khalifa@vet.usc.edu.eg; 3 Department of Clinical Pathology, Faculty of Veterinary Medicine, University of Sadat City, Sadat City 32897, Egypt; ahmed.omran@vet.usc.edu.eg; 4 Department of Pathology, Faculty of Veterinary Medicine, Cairo University, Cairo 12211, Egypt; reda_pathology@cu.edu.eg; 5 Department of Pathology, Faculty of Veterinary Medicine, Egyptian Chinese University, Cairo 11765, Egypt; 6 Department of Nutrition and Clinical Nutrition, Faculty of Veterinary Medicine, Menoufia University, Shibin El-Kom 32514, Egypt; 7 Department of Poultry and Fish Production, Faculty of Agriculture, University of Menoufia, Shibin El-Kom 32514, Egypt; eman.hussien@agr.menofia.edu.eg; 8 Department of Animal Production, College of Food and Agriculture Sciences, King Saud University, Riyadh 11451, Saudi Arabia; ralhotan@ksu.edu.sa; 9 College of Life Sciences, Northwest A & F University, Yangling District, Xianyang 712100, China; anamayyoub@nwafu.edu.cn; 10 Department of Physiology, Faculty of Veterinary Medicine, University of Sadat City, Sadat City 32897, Egypt; shaimaa.masoud@vet.usc.edu.eg

**Keywords:** thioacetamide, cardiotoxicity, silymarin, microalgae, histopathology, immunohistochemistry, oxidative stress, rats

## Abstract

This study assessed the possible pharmacological effects of *Chlorella vulgaris* (Cg), *Spirulina platensis* (St), and silymarin (Sl) against thioacetamide (TA)-induced cardiotoxicity in rats, with a focus on their antioxidant, cardioprotective, and anti-inflammatory properties. The following is the random grouping of sixty male rats into six groups of ten animals each: the control (negative control), TA-intoxicated group (positive control; 300 mg/kg body weight (BW)), Sl + TA group (100 mg Sl/kg BW + TA), St + TA group (400 mg St/kg BW + TA), Cg + TA (400 mg Cg/kg BW + TA), and St + Cg + TA group (400 St + 400 Cg mg/kg BW + TA) were all administered for 30 days. At the start of the study, groups 2 through 6 were administered TA intraperitoneally at a dosage of 300 mg/kg BW for two consecutive days, with a 24 h gap between each dose, to induce cardiac damage. Blood samples were obtained to measure hematological parameters and perform biochemical assays, including lipid profiles and cardiac enzymes. For histopathology and immunohistochemistry determination, tissue samples were acquired. The current findings showed that TA injection caused hematological alterations and cardiac injury, as evidenced by greater serum levels of troponin I, creatine kinase-MB, and total creatine kinase (*p* < 0.05), as well as significantly elevated serum malondialdehyde and decreased serum total antioxidant capacity (*p* < 0.05) concentrations. Moreover, an increase in blood low-density lipoprotein and total cholesterol concentration (*p* < 0.05) was recorded in the TA group. There were alterations in the heart tissue’s histological structure of the TA group compared to the control ones. These alterations were characterized by vacuolar degeneration of myocytes, loss of cross striation, coagulative necrosis, and fibrosis of interstitial tissue, which was ameliorated by the supplementation of SI, St, and Cg. The TA-intoxicated group showed weak expression of B-cell lymphoma protein 2 (*p* < 0.05) and strong immunoreactivity of tumor necrosis factor-α and B-cell lymphoma protein 2-associated X (*p* < 0.05). However, the groups receiving Sl, St, and Cg experienced the opposite. The administration of Sl, St, Cg, and St + Cg along with TA significantly improved and restored (*p* < 0.05) erythrogram indices, including RBCs, hemoglobin, total leukocytic count, lymphocytes, and monocyte, to the normal control values. The administration of Sl, St, and Cg alleviated the cardiotoxicity caused by TA via reducing oxidative stress, inflammatory markers, and apoptosis in heart tissue. In summary, the current findings suggest that the treatment with Sl, St, and Cg was beneficial in ameliorating and reducing the cardiotoxicity induced by TA in rats.

## 1. Introduction

Acute and long-term exposure to hazardous chemicals has been associated with higher rates of significant harm to many organs [1]. Thioacetamide (TA) has been widely used in many industrial sectors as an organic solvent as well as in the manufacture of catalysts, stabilizers, polymerization inhibitors, electroplating additives, photographic agents, insecticides, crosslinking agents, rubber auxiliaries, and more [2]. TA has potential use in drug production in addition to its industrial and environmental uses. Interestingly, TA plays an important part in antithrombotic medications, including thiazole, a non-steroidal anti-inflammatory medicine, and nizatidine, a histamine antagonist [3]. Although TA is well known for its severe hepatotoxicity, experimental research has shown that animals exposed to TA also exhibit brain dysfunction and renal epithelial damage [2,3]. Likewise, previous findings have shown that exposure to TA can have various harmful consequences on the heart, bones, bone marrow, and digestive system [3]. Studies have demonstrated that short-term TA injections cause negligible cardiac injury in experimental animals [4]. The concentration of TA and the body’s metabolic systems’ neutralization of harmful metabolites are two possible explanations for this phenomenon. Nonetheless, more recent studies have shown that giving experimental animals larger doses of TA over longer periods might cause cardiovascular injury [2]. Because of the role played by gut microbiome Toll-like receptor (TLR) signaling, there is a strong association between the activation of vascular endothelial cells and the development of acute liver failure brought on by TA [5]. This complex interaction emphasizes how important vascular endothelial cell activation is to the pathophysiology of acute liver failure [5]. Additionally, lipid peroxidation, production of reactive oxygen species (ROS), and detrimental effects on the antioxidant–oxidant system are the hallmarks of heart injury [2,3]. Certain studies indicate that oxidative stress plays a major role in the toxicity induced by TA [2,3]. Therefore, several antioxidant therapies have been suggested to reduce oxidative stress [6,7]. Recently, plant-based remedies have gained importance alongside more contemporary techniques. Technology’s advancement and the harmful side effects of pharmaceutical drugs used in medical treatment have raised interest in nutraceuticals and made it possible to study the bioactive substances of these plants.

One of the novel approaches to treating and preventing cardiovascular disease is including algae supplements in foods [8]. Algae is a significant source of novel therapeutic medicinal compounds. Cardio-protective substances are among the main and well-established uses of algae components. Microalgae are one of the best sources of polysaccharides, proteins, vitamins, phycobiliproteins, carotenoids, and sterols [9]. *Chlorella vulgaris* (Cg) contains 45% protein, 20% fat, 20% carbohydrates, 10% minerals and vitamins, and 5% fiber [10]. In addition to protein, fat, minerals, and other vitamins, Cg is a rich source of antioxidants, including vitamins C and E, polyphenols, omega-3 and 6, docosahexaenoic acids, eicosatetraenoic, lycopene, lutein, and carotenoids [9,10]. As a result, there is growing interest in using this supplement to control the lipid profile, blood sugar level, inflammation, and oxidative stress. Certain studies suggest that administrating Cg may help reduce inflammation and oxidative damage [10]. Supplemental Cg has immune-modulating, antioxidant, and lowering effects on blood sugar and cholesterol levels; thus, it has been suggested to have cardioprotective effects [10,11]. The cardioprotective effect of cg was documented to be owing to its antihypertensive properties through the modulation of endothelial function, noradrenalin, nitric oxide release, and anti-inflammatory and antioxidant properties [11].

St is a naturally occurring blue-green spiral filamentous alga that has been shown to have a high nutritional value and elevated antioxidant capacity [12]. Numerous investigations have shown that St offers therapeutic benefits, including anti-inflammatory, hypolipidemic, antioxidant, antidiabetic, and brain-protective properties [13,14,15,16]. Remarkably, several studies revealed that St contains various natural pigments, including C-phycocyanin, diadinoxanthin, diatoxanthin, and β-carotene, that have strong scavenging activity [13,14,15]. The benefits of *Spirulina* on a range of cardiovascular and cerebrovascular diseases are ascribed to its capacity to avert or mitigate cardiovascular risk factors, including hypertension, hyperglycemia, and hyperlipidemia [15], because of its antioxidant potential. The antioxidant activity of St could be due to its high quantity of phenolic hydroxyl groups, which is responsible for its capacity to scavenge radicals. Antioxidants, including polyphenols, have redox properties that allow them to quench singlet and triplet oxygen, reduce proxygene, and absorb and neutralize free radicals [16]. Additionally, supplementing with *Spirulina* has led to a reduction in endothelial damage markers, as well as an increase in glutathione peroxidase activity and levels of oxidized glutathione, indicating that *Spirulina* has the potential to improve endothelial function and that its antioxidant merits may help reduce cardiovascular problems [15].

Silymarin (Sl), *Silybum marianum* L. Herb, known in English as Mariendistel and Mary Thistle, is a unique herb found in Germany [17]. Silymarin consists of a mixture of four flavonolignans, silibinin (silybin A, B, and iso silybin A and B), isosilichristine, silychristine, silydianine, and one flavonoid, taxifolin, and silymarin, which are among the five main compounds of Sl. Silybin is the main component, approximately 60% to 70%, and it is believed to have the utmost biological activity. Sl has hepatoprotective and antioxidant activities since it can prevent the production of free radicals when toxic compounds are metabolized [18]. It has been observed that Sl shields rat cardiac tissues from oxidative stress and myocardial infarction brought on by ischemia–reperfusion through a variety of mechanisms, including anti-inflammatory, enhanced antioxidant defense systems, free radical scavenging, membrane stabilization, iron-chelating action, and prevention of apoptosis [19], thereby preventing the loss of membrane integrity and maintaining the membrane’s stability.

Hence, our study aimed to evaluate the cardioprotective effect of Sl, St, and Cg on TA-induced cardiotoxicity in male rats by determining changes in cardiac histopathology, cardiac enzymes, blood biochemical analyses, and immunohistochemistry of inflammatory markers. We hypothesized that these nutraceuticals could induce cardioprotective effects through their antioxidant and anti-inflammatory properties.

## 2. Materials and Methods

### 2.1. Materials

Thioacetamide (TA) was provided by The SDFCL company, India (CASR NO (62-55-5), with a purity of 99%. The sample SOP number for this product was SDFCL-TAR-SWP-GEN-007. Silymarin (*Silybum marianum* L.) was obtained from the Faculty of Pharmacy, Tanta University, Egypt. The phytochemical composition of Sl is presented in Table 1. St and Cg were obtained as a pure powder from the Algal Biotechnology Unit, National Research Centre (Giza, Egypt). The phytochemical composition of Cg was recently published by El-Gendy et al. [20]. The phytochemical composition of St is shown in Table 2. The required daily dose of algae is dissolved in water to obtain a suspension form on the day of administration to rats using an ultrasonic homogenizer (Biologics Inc., Manassas, VA, USA) according to El-Gendy et al. [20]. The total phenolic and flavonoid contents of St and Cg were accomplished following the Folin–Ciocalteu method [21] and the method of Kim et al. [22], respectively. Phenolic and flavonoid compounds of St and Cg extract were performed using an HPLC apparatus (Agilent Series 1100, Agilent, VA, USA) according to the methods of Lin et al. [23] and Kuntic et al. [24]. The HPLC system comprised two LC-pumps (series 1100), an auto-sampling injector, a solvent degasser, a UV/Vis detector (tuned at 250 nm for phenolic acids and 360 nm for flavonoids), and ChemStation software (version 11). C18 column (125 mm × 4.60 mm, 5 µm particle size) was used for the study. Using a gradient mobile phase consisting of two solvents, Solvent A (methanol) and Solvent B (acetic acid in water; 1:25), phenolic acids were separated. For the first three minutes of the gradient program, 100% B was the concentration. Eluent A was then added at a rate of 50% for the next five minutes. Then, for the next two minutes, the concentration of A was raised to 80%, and for the next five minutes, the detection wavelength was set at 250 nm. Using an isocratic elution (70:30) procedure, flavonoids were separated using a mobile phase consisting of two solvents: acetonitrile (A) and 0.2% (*v*/*v*) aqueous formic acid (B). The separation was carried out at a temperature of 25 °C with a solvent flow rate of 1 mL/min. There were 25 μL injection volumes.

### 2.2. Rats and Trial Design

Sixty male albino Wistar rats, weighing 156.7 g ± 3.4 g (mean ± SE), were acquired from the Laboratory Animal Colony (Giza, Egypt). Rats received an appropriate meal (AL Majd Company, Egypt) and ad libitum water and were kept in basic sanitary conditions in polypropylene cages during the trial. The temperature was maintained at 20–25 °C with a 12 h light/dark cycle and regular ventilation. Animals were acclimated for two weeks before the start of the study. All experimental procedures and methods were approved by the Research Ethics Committee of the Faculty of Veterinary Medicine, University of Sadat City, Egypt. Animal care complied with the guidelines and followed the Animal Care House’s rules (VUSC-018-1-24). Animals were randomly allocated into 6 groups of 10 rats each, as shown in Table 3. The experimental duration was 30 days.

Group 1, the control negative group, received distilled water orally for 30 days and normal saline at a dose of 1 mL intraperitoneally (IP) on the first two days of the experiment. Group 2, the TA group, a control positive group, was intoxicated with TA in a dose of 300 mg/kg body weight (BW) during the first two days of the experiment [25]. Group 3, the Sl + TA group, received Sl orally in a dose of 100 mg/kg BW for 30 days and TA IP in a dose of 300 mg/kg BW during the first two days of the experiment [26]. Group 4, the St + TA group, was given St in a dose of 400 mg/kg BW for 30 days and received TA IP at a dose of 300 mg/kg BW during the first two days of the experiment [27]. Group 5, the Cg + TA group, received Cg orally at a dose of 400 mg/kg BW per day for 30 days and TA IP in a dose of 300 mg/kg BW in the first two days of the experiment [28]. Group 6, the St + Cg + TA group, received St and Cg orally at doses of 400 + 400 mg/kg BW for 30 days and TA IP at a dose of 300 mg/kg BW in the first two days of the experiment.

### 2.3. Sampling

In this experiment, a weight measuring scale was used to weigh the rats before the start of the trial and on the day of scarification. The body gain was calculated from the difference between the body weight at the beginning and the end of the experiment. Rats were starved for an entire night after the end of the trial to obtain blood samples. Five randomly chosen rats were given light ether anesthesia (Sigma Chemical Co., St. Louis, MO, USA), and blood samples were obtained from retro-orbital bleeding. Each blood sample was divided into three portions. The first portion was collected for hematological tests in a tiny, clean, labeled vial that contained EDTA. After the second portion of blood was collected, it was centrifuged at 3000 rpm for 15 min to separate the sera in plain, dry, sterile, and non-heparinized centrifuge tubes. The tubes were then allowed to clot. Before being subjected to biochemical analysis, the acquired sera samples were collected and stored individually in dry, clean bottles in a deep freezer at −20 °C. Once blood samples were taken, rats decapitated their necks to obtain a heart sample, and the heart samples were then preserved in 10% neutral formalin solution for immunohistochemical and histological investigations.

### 2.4. Hematological Analysis

Using an automated hematology analyzer and blood cell counter (Sysmex F-800, Tokyo, Japan), the whole blood samples were used immediately upon collection to estimate the following hematological parameters, including hemoglobin (Hb) concentration, red blood cells (RBCs), hematocrit value (PCV%), total leucocyte count (TLC), platelet counts (Plt), and differential leukocyte counts [29].

### 2.5. Biochemical Assay

The biochemical parameters of cardiac tissue indicators were determined in the collected serum samples following the manufacturer’s guidelines. Total creatine kinase (CK) and creatine kinase MB (CK-MB) for myocardial muscle (CK- MB, ThermoFisher Scientific, Waltham, MA, USA, Elisa Kit) were assessed according to Aujla and Patel [30]. Troponin I was estimated according to Jiang et al. [31] using an available commercial kit (Troponin, ThermoFisher Scientific, Waltham, MA, USA, Elisa Kit) and following the kit manufacturer’s instructions. Lipid profile, including total cholesterol (TC), triglyceride (TG), high-density cholesterol (HDL), and low-density cholesterol (LDL), was determined following Rahmani et al.’s [32] protocols.

### 2.6. Evaluation of Oxidant/Antioxidant Biomarkers

Oxidative stress is revealed by measuring total antioxidant capacity (TAC) using commercial kits (TA2513, ELISA kit, Biodiagnostic Co., Giza, Egypt), as described by Koracevic et al. [33]. Malondialdehyde (MDA), a marker of lipid peroxidation, was assessed using commercial kits (Cat No. ab118970, Abcam Co., Cambridge, UK) following the manufacturer’s instructions.

### 2.7. Histopathological Examination

Heart tissue samples were taken from the experimental groups, preserved in 10% neutral-buffered formalin, cleaned, dried, and embedded in paraffin. Hematoxylin and Eosin (H&E) were used to stain the paraffin-embedded blocks after they were sectioned at a thickness of five microns [34]. Cardiac tissue lesions were graded as having no changes (0), mild changes (1), moderate changes (2), and severe alterations (3). The grading was then calculated as a percentage as follows: mild changes, moderate changes, and severe changes [35].

### 2.8. Immunohistochemistry

The immunohistochemistry procedure was performed using the guidelines provided by Shaaban et al. [36]. After being deparaffinized in xylene, tissue pieces were rehydrated in various alcohol grades. The sections were pretreated with a pH 6 citrate buffer for 20 min to retrieve the antigen. Rabbit polyclonal anti-Bcl-2 antibody at a concentration of 1:50 (ab59348; Abcam, Cambridge, UK), rabbit monoclonal anti-Bax antibody [E63] at a concentration of 1:250 (ab32503; Abcam, Cambridge, UK), and anti-TNF-α (ab270264; 1:100 dilution rate, Abcam, Cambridge, UK) were incubated on sections for two hours in a humidified chamber. Goat anti-rabbit IgG H&L (HRP; ab205718; Abcam, Cambridge, UK) was employed as the chromogen for the sections’ incubation, and 3,3′-diaminobenzidine tetrahydrochloride (DAB, Sigma) was the source of the antibody. The slides were then mounted with DPX and counterstained with hematoxylin. To make the negative control slides, PBS was used to replace the primary antibodies. Each group’s five tissue slices had five different Bax, Bcl-2, and TNF-α quantitative immunoreactivities assessed. Using a high-power microscopic field (X 400), immunoreactivity was examined in ten microscopical fields per section. Color deconvolution picture J 1.52 p software (Wayne Rasband, National Institutes of Health, Bethesda, MD, USA) was used to estimate the percentage of positively stained cells (%) [37].

### 2.9. Statistical Analysis

Data were checked for normality by the Kolmogorov–Smirnov and Levene’s tests before statistical analysis. Data were subjected to a one-way ANOVA using IBM SPSS software version 21, and Duncan’s multiple comparison tests determined significant variations between the treatment groups (*p* < 0.05). The experimental unit was the rat for all measurements. Statistical significance was defined at a probability of *p* < 0.05, and values were presented as means ± standard error (SE).

## 3. Results

### 3.1. Animals Health Condition, Body Weight, and Weight Gain

**Figure 1:**
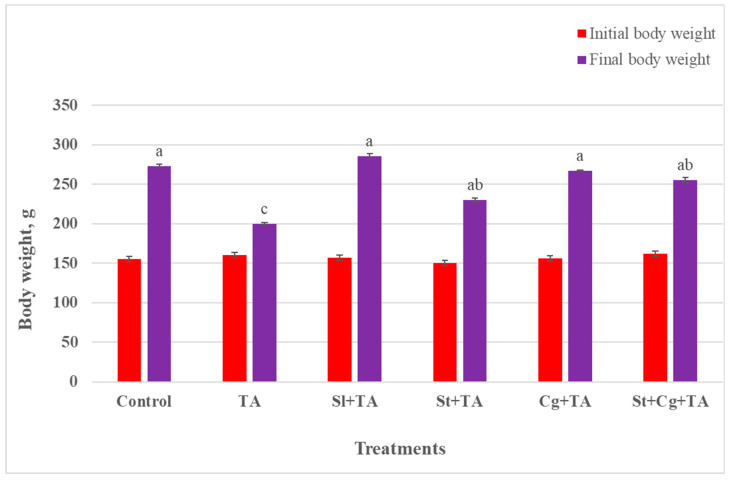
Effect of TA, Sl, St, and Cg on final body weight (g) of the experimental rats. Values are represented as means ± SE, *n* = 10. Different letters (a, b, c) indicate statistical significance at *p* < 0.05. TA, thoiacetamide; Sl, silymarin; St, *Spirulina platensis*; Cg, *Chlorella vulgaris*.

**Figure 2:**
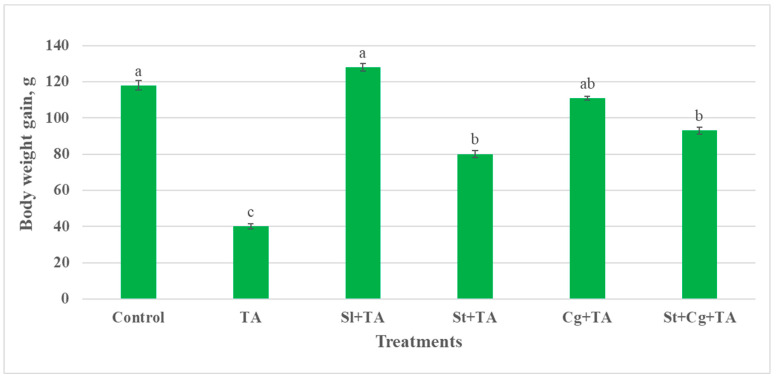
Effect of TA, Sl, St, and Cg on body weight gain (g) of the experimental rats. Values are represented as means ± SE, *n* = 10. Different letters (a, b, c) indicate statistical significance at *p* < 0.05. TA, thoiacetamide; Sl, silymarin; St, *Spirulina platensis*; Cg, *Chlorella vulgaris*.

Rats of the control, Sl-, St-, and Cg-treated groups did not show any clinical manifestations. Conversely, dullness, depression, reduced feed intake, lethargy, rough hair coat, and dehydration were the observed signs of the TA-treated group. BW and body gain showed a significant decrease in the TA-treated group (*p* < 0.05) in comparison to the control and the other treatment groups (Sl + TA, St + TA, Cg + TA, and St + Cg + TA) (Figure 1 and Figure 2).

### 3.2. Blood Indices

The effects of the administration of Sl, St, Cg, and St + Cg with TA toxicity on erythrogram indicators are presented in Table 4. In contrast to the TA group, the supplementary groups could return the RBC and Hb levels to normal values (*p* < 0.001). On the other hand, there was a non-significant difference in PCV, MCV, MCHC, MCH, and PLTs among the treatment groups. Rats given TA had significantly lower erythrogram indices (*p* < 0.001) than the control group. Additionally, the TA-treated group had significantly higher neutrophil and monocyte counts than the other groups’ mean values (*p* < 0.05). In contrast, the control positive group had lower lymphocyte counts than the other groups’ mean values (*p* < 0.05). There were significant increases (*p* < 0.01) in TLCs and lymphocyte levels between the Sl, St, Cg, and St + Cg with TA toxicity groups and the TA group. However, rats intoxicated with TA exhibited a significant increase (*p* < 0.05) in N/L ratio compared to those of the control group and other groups (*p* < 0.05).

### 3.3. Cardiac Enzymes

The estimated concentrations of the three cardiac enzymes (total CK, CK-MB, and troponin I) in the experimental groups are displayed in Table 5. When comparing the TA-intoxicated group to the control group, there was a substantial (*p* < 0.05) increase in serum total CK, CK-MB, and troponin I activity. The total CK, CK-MB, and troponin I activity decreased to nearly normal levels in the Sl-, St-, Cg-, and St + Cg-treated groups compared to the control group.

### 3.4. Serum Lipid Profile

The effect of the administration of Sl, St, Cg, and St + Cg with TA toxicity rats on the serum lipid profile is presented in Table 6. There was a substantial rise in total cholesterol and LDL in the TA-intoxicated group compared to the control and other treatment groups (*p* < 0.05). In contrast, there was no difference in the treatment groups’ serum concentrations of triglycerides or total lipids. The TA-intoxicated group showed a significant decrease (*p* < 0.05) in the HDL level compared to the control and other treatment groups. Serum HDL concentrations were decreased in all treatment groups (*p* < 0.001) compared to the control group, with the TA-treated group exhibiting a more marked decline.

### 3.5. Serum Protein Levels

The effect of Sl, St, Cg, and St + Cg administration with TA toxicity on the serum protein profile of rats is shown in Table 7. Although there was a significant increase in total protein and globulin in the Sl-, St-, and Cg-treated groups when compared with the control and TA groups, there was a decrease in total protein, albumin, globulin, and A/G ratio in the TA-intoxicated group when compared with the control group. Additionally, the Sl-, St-, and Cg-treated groups had a lower A/G ratio compared with the control group (*p* < 0.01).

### 3.6. Serum Oxidant/Antioxidant Biomarkers

Table 8 shows the effects of Sl, St, Cg, and St + Cg treatment on serum oxidant/antioxidant indicators in rats exposed to TA toxicity. Serum TAC dramatically dropped in the TA-intoxicated group even though their MDA concentration was greater than that of the control and other treatment groups. The Sl-, St-, and Cg-treated groups showed a significant increase in TAC and a significant decrease in MDA (*p* < 0.05) in comparison to the TA-intoxicated group.

### 3.7. Histopathological Findings

**Figure 3:**
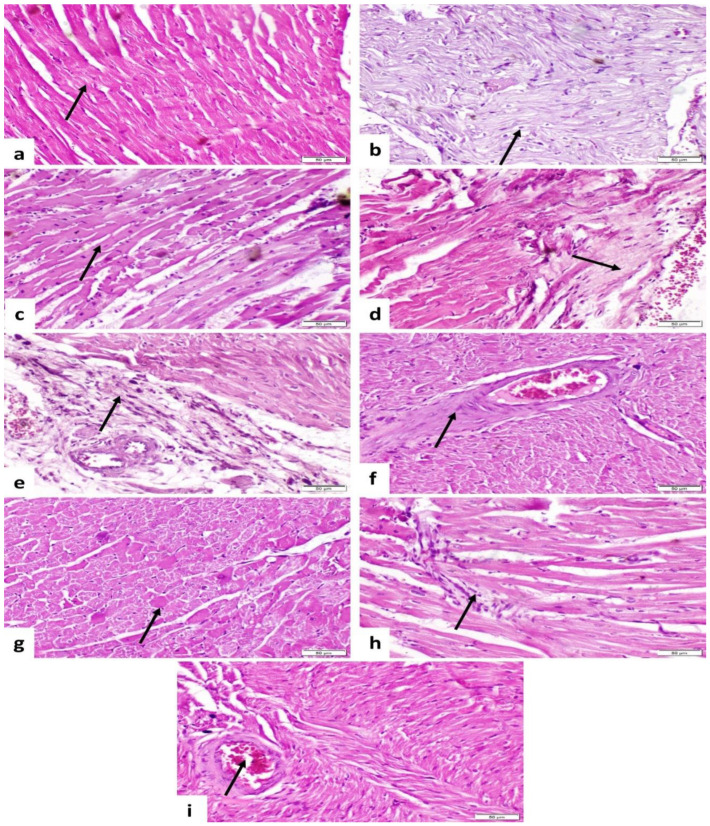
Photomicrograph of rat heart (H&E-stained sections, scale bar 50 µm): (**a**) the control group showed normal histological structure of myocytes (arrow). (**b**) The group treated with TA showed vacuolar degeneration of myocytes (arrow). (**c**) The group treated with TA showed loss of cross striation and sarcoplasm was more eosinophilic (arrow). (**d**) The group treated with TA showed fibrosis of interstitial tissue (arrow). (**e**) The group treated with TA showed perivascular fibrosis and inflammatory cell infiltration. (**f**) The group treated with TA showed hypertrophy of the tunica media of blood vessels. (**g**) The SI + TA group showing necrosis of few myocytes (arrow); (**h**) the SI + TA group showing mild fibrosis and inflammatory cells infiltration (arrow); (**i**) the SI + TA group showing normal interstitial blood vessel (arrow).

**Figure 4:**
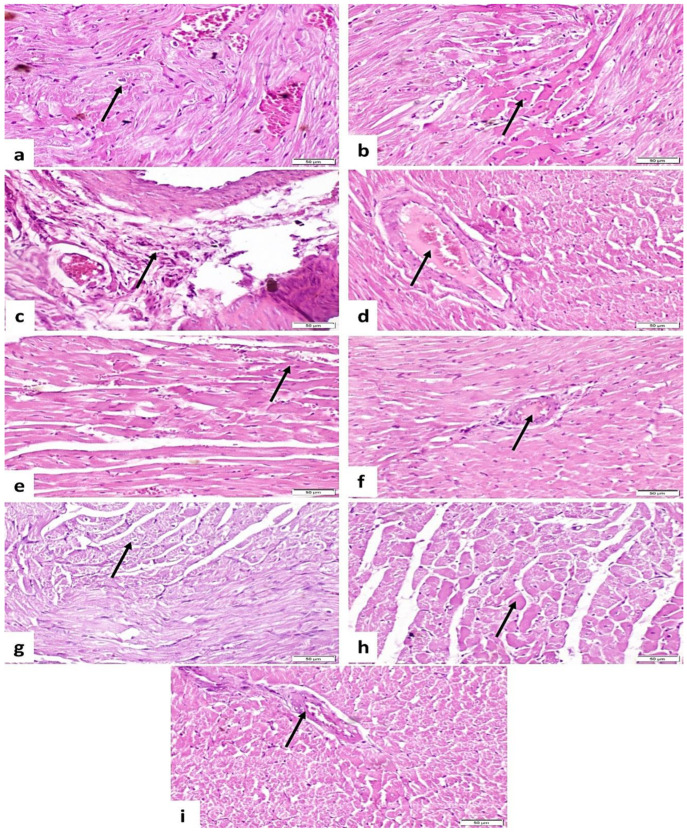
Photomicrograph of rat heart (H&E-stained sections, scale bar 50 µm): (**a**) the St + TA group had vacuolar degeneration of myocytes (arrow). (**b**) The St + TA group showed necrosis of myocytes (arrow). (**c**) The St + TA group showed perivascular fibrosis and inflammatory cell infiltration (arrow). (**d**) The St + TA group showed a mild thickness of interstitial blood vessels (arrow). (**e**) The Cg + TA group had a necrosis of a few myocytes (arrow). (**f**) The Cg + TA group had normal blood vessel thickness (arrow). (**g**) The St + Cg + TA group had mild vacuolar degeneration (arrow); (**h**) the St + Cg + TA group showed mild necrosis (arrow); (**i**) the St + Cg + TA group showed normal vascular thickness (arrow).

Concerning the control group, the heart showed normal histological structure (Figure 3a). The group treated with TA showed vacuolar degeneration of myocytes (Figure 3b) and also revealed Zenker’s necrosis of myocytes (loss of cross striation and sarcoplasm was more eosinophilic) (Figure 3c), fibrosis of interstitial tissue (Figure 3d), perivascular fibrosis and mononuclear inflammatory cells infiltration (Figure 3e), and thickening and hypertrophy of tunica media of interstitial blood vessels (Figure 3f). The SI + TA group showed necrosis of a few myocytes (Figure 3g), mild fibrosis, inflammatory cell infiltration (Figure 3h), and normal interstitial blood vessels (Figure 3i). The St + TA group showed mild improvement as vacuolar degeneration and necrosis of myocytes were moderate (Figure 4a,b); also, there was perivascular fibrosis, mononuclear inflammatory cells infiltration (Figure 4c), and mild thickness of interstitial blood vessels (Figure 4d). The Cg + TA group revealed necrosis of a few myocytes (Figure 4e) and nearly normal interstitial blood vessel thickness (Figure 4f). The St + Cg + TA group showed a noticeable amelioration, vacuolar degeneration, and mild necrosis (Figure 4g,h) with a normal vascular thickness (Figure 4i). Lesions in heart tissue were recorded and scored according to severity as shown in Table 9.

### 3.8. Immunohistochemical Findings of Bax, Bcl-2, and TNF-α in Heart

**Figure 5:**
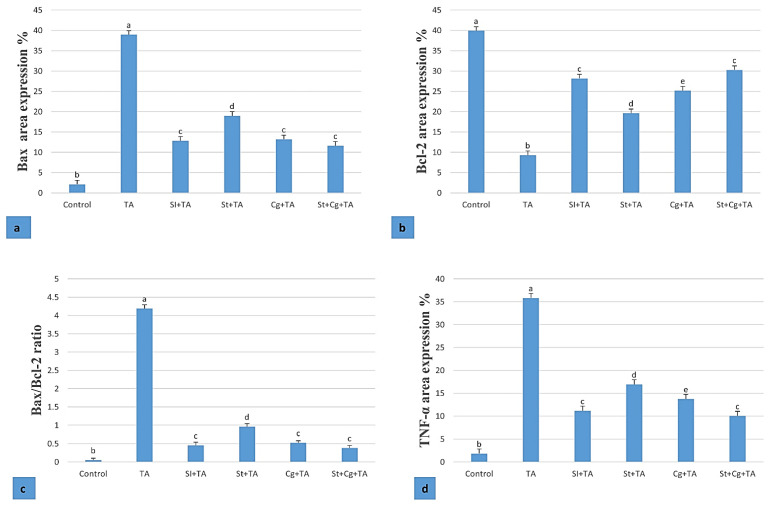
(**a**) Immunostaining area expression % of BAX protein in the heart of different groups. (**b**) Immunostaining area expression % of Bcl-2 protein in the heart of different groups. (**c**) Immunostaining area expression % of TNF-α in the heart of different groups. (**d**) BAX/Bcl-2 ratio in experimental groups. Data were expressed as mean ±SE, with different letters indicating significant differences at *p* < 0.05.

**Figure 6:**
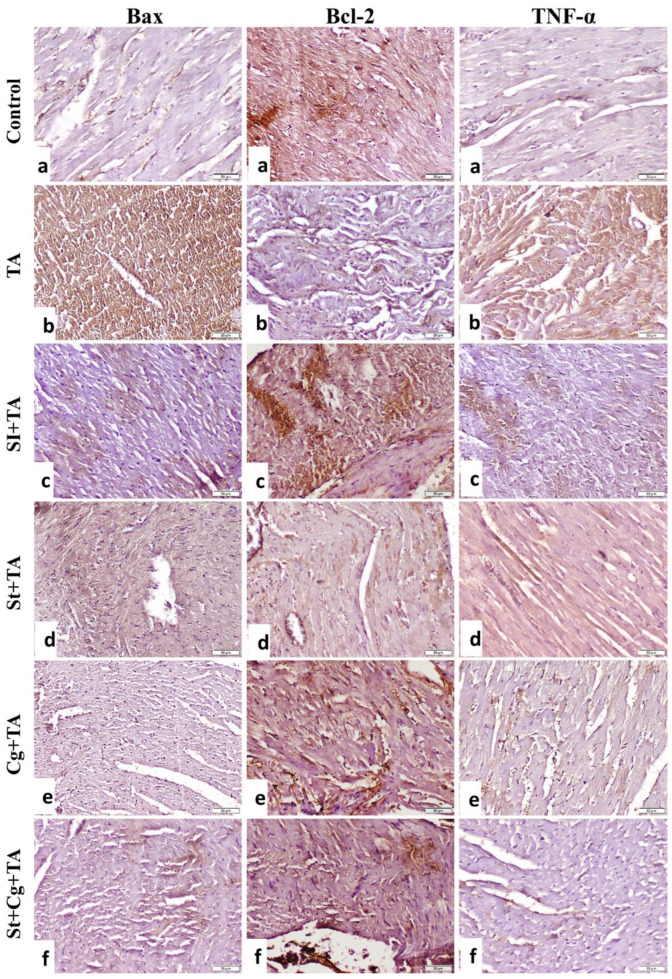
Photomicrograph of rat heart (Bax-, Bcl-2-, and TNF-α-stained sections, scale bar 50 µm): (**a**) the control group showed weak immune expression of Bax and TNF-α and strong expression of Bcl-2. (**b**) The TA group showed strong immunoreactivity of Bax and TNF-α and weak expression of Bcl-2. (**c**) The SI + TA group showed weak expression of Bax and TNF-α and strong expression of Bcl-2. (**d**) The St + TA group showed moderate expression of Bax, Bcl-2, and TNF-α. (**e**) The Cg + TA had mild expression of Bax and TNF-α and strong expression of Bcl-2. (**f**) The St + Cg + TA group had mild expression of Bax and TNF-α and strong expression of Bcl-2.

The immunostaining expression of Bax, Bcl-2, and TNF-α area % in the heart tissue and Bax/Bcl-2 ratio are presented in Figure 5. The control group showed feeble immune expression of Bax and TNF-α and strong expression of Bcl-2 (Figure 6a) compared to other groups. The TA group showed strong immunoreactivity of Bax and TNF-α and weak expression of Bcl-2 (*p* < 0.05) compared to the control and other treatment groups (Figure 6b). The Sl + TA group showed weak expression of Bax and TNF-α and strong expression of Bcl-2 (Figure 6c). The St + TA group showed moderate expression of Bax, Bcl-2, and TNF-α (Figure 6d). The Cg + TA and St + Cg + TA groups showed mild expression of Bax and TNF-α and strong expression of Bcl-2 (Figure 6e,f).

## 4. Discussion

The basis for the toxic effect of TA on tissues, including the liver, the heart, the kidney, and the brain, is the initiation of oxidative stress, ROS production, and inflammation [2,3]. Our study investigated the cardioprotective benefits of Sl, St, and Cg against TA-induced cardiotoxicity via modulating the antioxidant, anti-inflammatory, and immunological activities of Sl, St, and Cg.

Our findings revealed a decline in BW and weight gain of rats who received TA, which may be due to the direct toxic influences of TA. The observed decrease in BW and weight gain in rats given TA was most likely caused by malnutrition brought on by the reduction in appetite, food intake and absorption, and gastrointestinal toxicity [38,39]. Also, it could be accredited to renal damage causing much loss of water, proteins, and salts, which results in weight loss and dehydration [40]. In this study, Sl, St, and Cg improved BW and weight gain that TA induced; these results are in line with those of Abd El-Ghany [38] who reported that Sl increased BW due to an enhanced gastrointestinal health condition. On the other hand, St + TA treatment reduced BW and weight gain compared to the control group, but it performed better than the TA-intoxicated group. Our results are consistent with those of DiNicolantonio et al. [40] and Sanayei et al. [41], who found that St and Cg decreased BW and weight gain because of a decrease in body fat mass and lipid profile, respectively.

The reduction in RBCs count in the TA rats may be ascribed to the action of TA on a hematopoietic approach, which is damaged through contact with TA and reduction in Hb concentration that results in an elevated demolition of RBCs or drop off in the expanse of RBCs synthesis. IP injection of TA induced acute cardiac injury with a decrease in the values of Hb, RBCs, and PCV [42]. Neutrophilia and lymphocytopenia were observed in the TA-treated group [43]. This might be explained by a weakening of the immune system brought on by tissue damage and acute toxicity that could be linked to TA exposure. The results of the current trial revealed that Sl, St, and Cg supplementation lessened the changes in hematological parameters caused by TA toxicity. These findings were consistent with Karagül et al. [44], who found that Sl improved blood parameters. Sl is rich in Vit. C and iron, which increase immunity and hematopoiesis levels, as reported by Khazaei et al. [45]. Furthermore, Abdel-Aziz et al. [46] reported that oral administration of St and Cg increased Hb, RBCs, PCV, and platelet values. This may allude to the possibility that St plays a significant role in erythropoiesis [46]. Furthermore, Cg has been shown to improve hemato-biochemical parameters [47]; this may be related to the proteinaceous components it has in its constituents, which can improve red blood cells. St and Cg are rich sources of nutrients, especially protein and essential amino acids [48], and are considered ironic plants, which have been documented to boost blood parameter levels [49].

In the present study, TA-treated rats showed a significant decrease in TP, albumin, and globulin levels with an increase in the A/G ratio compared to the other treatment groups. This finding is in line with Megahed et al. [50], who reported that TA resulted in a significant decline in TP, albumin, and globulin because oxidative stress and ROS produced by TA attack proteins, lipids, and DNA. This leads to hepatocyte damage, thus reducing TP and albumin production from hepatocytes [51]. Conversely, Sl, St, and Cg supplementation significantly improved TP, globulin, and albumin with a significant decrease in the A/G ratio when compared with those of the TA-treated group. Similarly, Eid et al. [52] found that Sl improved the levels of TP, globulin, and albumin in laying hens due to the fact that Sl improved liver health condition to produce TP. Ouedraogo et al. [53] stated that St resulted in an increase in TP and globulin with significant decreases in the A/G ratio and albumin. Also, Rahman et al. [54] reported that St significantly improved TP, globulin, and albumin with a reduction in the A/G ratio in catfish because St had a hepatoprotective effect and improved the health state of hepatocytes. In addition, Cg enhanced TP, albumin, and globulin in Tilapia fish due to improved liver function [55].

Compared to the liver, heart tissue is more susceptible to injury from ROS because it has a lower antioxidant defense and a greater rate of oxidative metabolism [19]. Moreover, given the role played by gut microbiome TLR signaling, there is a strong relationship between the stimulation of vascular endothelial cells and the development of acute liver failure brought on by TA [5]. This complex interaction highlights how important vascular endothelial cell activation is to the pathophysiology of acute liver failure [5]. TA induced cardiac and multi-organ damage, which is characterized by elevated TBARS levels and raised ROS production, which in turn are detrimental to cellular components such as proteins, lipids, and DNA. It can also affect the structure and functionality of cells [2,25]. In the current trial, TA administration caused a substantial elevation in serum cardiac enzyme (total CK, CK-MB, and troponin I) activities and MDA concentration but decreased serum TAC. These results are consistent with those reported by Kundu et al. [56], and they attributed these effects to the toxic organo-sulfur material of TA which is quickly converted to reactive components and ROS [25]. On the other hand, St, Cg, and Sl reduced serum levels of cardiac enzymes and MDA to nearly the normal values while increasing TAC levels. Singh et al. [57] found that Sl decreased cardiac enzymes such as troponin I, CK-MB, and total CK, which were elevated as a result of doxorubicin administration, suggesting the cardioprotective and antioxidant effect of Sl. Mirzaei et al. [58] observed that Sl improved Thiol and TAC, markers of antioxidant capacity, and decreased the MDA serum level. Phytochemicals of Sl were reported to neutralize the cytotoxic free radicals produced during ischemia–reperfusion injury, and Sl can preserve and stabilize the membrane, preventing it from losing its integrity [59], thereby shielding myocytes from oxidative damage. Sl contains flavonolignans (silybin A, B, silychristin, isosilybin A, B, silydianin, isosilychristin, and the flavonoids quercetin, taxifolin, and kaempferol) as its main ingredients, which have antioxidant capabilities [60].

Furthermore, St improved cardiac tissue and decreased cardiac enzymes such as troponin I and CK-MB in rats, as reported by Albtoosh et al. [61], because St guards the reliability of cardiac myocytes after being handled by TA. Attia et al. [62] found that St enhanced the level of TAC; in contrast, the level of MDA was decreased. St is a unique and concentrated source of nutrients that includes minerals, γ-linolenic acid, phycocyanin, vitamin E, β-carotene, proteins, and B-complex vitamins. Most of these elements have extremely high antioxidant potential and radical scavenging abilities, providing a defense against oxidative stress [63]. Consequently, *Spirulina*’s micronutrients and antioxidant components help to mitigate the oxidative stress brought on by TA toxicity. Abdelbaky et al. [10] and Barghchia et al. [11] stated that the cardioprotective effect of Cg may be owing to its anti-inflammatory and antioxidant effects. Moreover, Farag et al. [64] reported that Cg enhanced the TAC level but decreased the MDA concentration. Cg is a rich source of antioxidants that was reported to improve heart health status [11]. The antioxidant gift of Cg has been conveyed to its phenolic constituents identified with other functioning phytoconstituents, including lutein, catechins, caffeic acid, carotenoids, gallic acid, benzoic acid, rutin, and chlorogenic acid [11]. In the present study, the TA-treated rats showed a significant increase in TC and LDL and a significant decrease in HDL; these results are in line with Ebaid et al. [65], who reported that TA increased TC and TG in rats. Blood lipid markers aid in monitoring cardiovascular health. Greater levels of LDL have been correlated with an excessive risk of atherosclerosis, while a raised level of HDL is associated with a decreased incidence of cardiovascular diseases [66]. In the present study, we noticed that Sl, St, and Cg supplementation improved the lipid profile with a decrease in TC and LDL levels and an increase in HDL levels. These findings are consistent with those of Mohammadi et al. [67], who found that Sl reduced TG, TC, and LDL and increased HDL to lower the risk of atherosclerosis and heart disease. Sl can influence lipid metabolism by diminishing hepatic cholesterol synthesis and restricting its absorption from the gastrointestinal tract [68]. Moreover, silybin found in Sl was reported to have hypolipidemic activity and its hypolipidemic property could be due to augmented endogenous cholesterol conversion to bile acids [69]. Rostami et al. [70] reported that St reduced LDL, TC, and TG, indicating the hypolipidemic effect of St because it contains γ-linolenic acid, necessary for prostaglandin production, which is beneficial and may influence several body activities, including the control of cholesterol synthesis. Karima and Sarto [71] claimed that Cg increased HDL levels while it decreased those of TC, TG, and LDL; this may be because Cg contains an omega-3 fatty acid, which completely compensates for the HDL shortage. Conversely, Cg was found to reduce LDL and TC levels while having no discernible effect on HDL or TG levels [72]. Furthermore, according to Deng and Chow [73], the antioxidant properties of St and Cg bioactive components may reduce the concentration of blood lipids because they lower pancreatic lipase activity, which sequentially reduces hepatic fatty acid synthesis. They may also lessen intestinal cholesterol absorption or synthesis [74]. However, the mechanism underlying these effects needs further research.

In support of the current biochemical findings, there were histopathological alterations, and lesion scores were promoted in cardiac tissues. TA-induced oxidative stress altered the architecture of the cardiac tissue, as confirmed by histopathological examinations and increased lesion scores. TA intoxication caused vacuolar degeneration of myocytes; it also revealed Zenker’s necrosis of myocytes, fibrosis of interstitial tissue, perivascular fibrosis, and mononuclear inflammatory cells infiltration and thickening, as well as the hypertrophy of the tunica media of interstitial blood vessels in rat cardiac tissues. As a result of heart injury, the altered permeability of the membrane causes the enzymes within the cells to be released into circulation, which damages the cardiac cells, as shown by the abnormally high level of serum cardio-specific enzymes. Our results agreed with previous studies [2,25]. On the other hand, administration of Sl, St, and Cg to rats intoxicated with TA reduced oxidative stress, which was evident in the histological alterations brought on by TA. This led to the recovery of the heart tissues’ original architecture and showed notable advancements in the reversal of these histological alterations. This result implies that one of the potential pathways in the pathophysiology of TA-induced heart tissue damage is thought to be the oxidative stress caused by the free radicals that are produced.

The immunohistochemical results, which showed overexpression of cardiac immunoreactivity of Bax and TNF-α and weak expression of Bcl-2 in TA-treated rats, were consistent with the current biochemical and histological results. However, the opposite of these histochemical markers was correctly noted in the Sl-, St-, and Cg-treated groups. Oxidative stress, caused by the increased production of ROS, is thought to be a key risk factor in the development of heart disease [75]. Production of a large quantity of ROS owing to TA can beat the antioxidant defense mechanism and distrust cellular components. *TNF-α* is a proinflammatory cytokine that is rapidly produced in response to tissue damage from macrophages. The histological evidence of myocardial necrosis has been closely associated with an increase in TNF-α immune expression. TNF-α worsens heart failure by disrupting the mechanism that preserves homeostasis, leading to imbalance and suppressing anti-inflammatory responses.

This investigation demonstrated a noteworthy rise in TNF-α immune expression after TA administration. Treatment with Sl reduced these modifications, suggesting that Sl either promotes or inhibits TNF-α degradation or secretion [60]. The active component of the main flavonolignans, silybins A and B, silychristin, isosilybins A and B, and silydianin, is typically linked to Sl’s anti-inflammatory properties. Therefore, it aids in the suppression of inflammation [76]. Chen et al. [77] observed that St and its active constituent, C-phycocyanin, lowered interleukin-6, Cyclooxygenase-2, TNF-α, and nitric oxide synthase, as well as reduced TNF-α release. According to Farag et al. [64], Cg inhibits the release of TNF-α, which may be explained by its antioxidant properties and ability to reduce reactive oxygen species (ROS), which are known to increase the levels of TNF-α and IL-1β.

Apoptosis pathways may be categorized into two paths: the intrinsic pathway, which is directed by mitochondria, and the extrinsic pathway, which is directed by death receptors [78]. Bcl-2 is a major protein tangled in the intrinsic pathway, amongst which Bax apoptosomes and activates caspase-3 to make apoptosis a pro-apoptotic protein when its expression level is elevated. Alternatively, as an anti-apoptotic protein, Bcl-2 inhibits Bax to stop apoptosis from progressing [79]. Eraky et al. [80] reported that TA increased the expression of Bax and caspase-8. The current trial proved a significant increase in Bax and a decrease in Bcl-2 and the Bax/Bcl-2 ratio after TA administration. These alterations were attenuated by Sl, St, and Cg supplementation, as previously reported [81,82,83]. The reason behind this effect might be the existence of polyphenols such as genistein, quercetin, and ellagic acid, which can significantly inhibit a variety of molecular targets, including apoptotic markers caspases, NF-κB, and Bcl and Bax [84]. However, the mechanism underlying these effects remains unclear and prompts further investigation.

## 5. Conclusions

In the current trial, TA injection caused hematological damage and cardiac injury, as evidenced by greater serum levels of troponin I, CK-MB, and total CK, as well as elevated serum MDA and decreased serum TAC concentrations. Moreover, an increase in blood LDL and TC concentration was recorded in the TA group. There were alterations in the heart tissue’s histological structure of the TA group compared to the control ones. These alterations were characterized by the vacuolar degeneration of myocytes, loss of cross striation, coagulative necrosis, and fibrosis of interstitial tissue, which was ameliorated by the supplementation of SI, St, and Cg. The TA-intoxicated group showed weak expression of Bcl-2 and strong immunoreactivity of TNF-α and Bax. However, the groups receiving SI, St, and Cg experienced the opposite. The administration of Sl, St, Cg, and St + Cg along with TA significantly improved and restored erythrogram indices, including RBCs, hemoglobin, total leukocytic count, lymphocytes, and monocyte, to the normal control values. The administration of Sl, St, and Cg alleviated the cardiotoxicity caused by TA by reducing oxidative stress, inflammatory markers, and apoptosis in heart tissue. The current findings suggested that supplementing with Sl, St, and Cg was advantageous in ameliorating cardiotoxicity caused by TA in rats. An in-depth analysis of alterations in the liver and gut microbiota should be assessed to investigate the hepato-cardiac axis, and the underlying molecular mechanism upon the beneficial effects of Sl, St, and Cg supplementation should be further evaluated.

## Figures and Tables

**Table 1:** HPLC fractions of phenolic and flavonoid contents of Sl.

Compounds	Concentration, µg/g
Gallic acid	9.96 ± 0.03
Neochlorogenic acid	7.73 ± 0.05
Luteolin	18.26 ± 0.01
Chlorogenic acid	6.29 ± 0.12
Apigenin	29.33 ± 0.05
Pinoquercetin	12.32 ± 0.03
3,3-dimethyl quercetin	19.56 ± 0.17
Methylquercetin	19.32 ± 0.03
Salviolinic acid	37.25 ± 0.44
Ferulic acid	4.02 ± 0.02
Rutin	24.62 ± 0.27
Total phenolics	1.7 mg gallic acid equivalent/g
Total flavonoids	7.43 mg quercetin equivalent/g

**Table 2:** HPLC fractions of phenolic and flavonoid contents of St.

Compounds	Concentration, µg/g
Gallic acid	11.25 ± 0.1
Neochlorogenic acid	6.72 ± 0.2
Luteolin	7.36 ± 0.35
Chlorogenic acid	5.45 ± 0.07
Apigenin	11.22 ± 0.29
Pinoquercetin	17.11 ± 0.06
3,3-dimethyl quercetin	24.25 ± 0.02
Methylquercetin	42.56 ± 0.25
Salviolinic acid	4.63 ± 0.01
Ferulic acid	4.56 ± 0.11
Rutin	2.33 ± 0.02
Total phenolics	1.58 mg gallic acid equivalent/g
Total flavonoids	8.63 mg quercetin equivalent/g

**Table 3:** The experimental design.

	Experimental Period (30 Days)	
Experimental Groups	On 1st and 2nd Day of the Experiment	Until the End of the Experiment
Control	Normal saline 1 mL was injected IP	Distilled water 1 mL
TA	300 mg/kg BW of TA was injected IP	……………………………
Sl + TA	300 mg/kg BW of TA was injected IP	100 mg/kg BW of Sl orally
St + TA	300 mg/kg BW of TA was injected IP	400 mg/kg BW of St orally
Cg + TA	300 mg/kg BW of TA was injected IP	400 mg/kg BW of Cg orally
St + Cg + TA	300 mg/kg BW of TA was injected IP	400 mg/kg BW of St orally + 400 mg/kg BW of Cg orally

TA, thoiacetamide; Sl, silymarin; St, Spirulina platensis; Cg, Chlorella vulgaris; BW, body weight, IP, intraperitoneal.

**Table 4:** Hematological parameters (hemogram and leukogram) of male rats.

Parameters	Treatments						*p*-Value
	Control	TA	Sl + TA	St + TA	Cg + TA	St + Cg + TA	
RBCs (10^6^/μL)	6.04 ± 0.16 ^ab^	5.89 ± 0.28 ^c^	6.24 ± 0.29 ^a^	6.40 ± 0.04 ^a^	6.31 ± 0.13 ^a^	6.15 ± 0.31 ^ab^	<0.001
Hb(g/dL)	13.23 ± 0.12 ^a^	11.36 ± 0.91 ^b^	13.56 ± 0.41 ^a^	13.33 ± 0.26 ^a^	13.23 ± 0.27 ^a^	13.06 ± 0.43 ^a^	<0.001
PCV (%)	32.16 ± 0.54	32.13 ± 1.37	32.93 ± 0.9	32.33 ± 0.54	31.96 ± 0.46	31.36 ± 0.09	<0.001
MCV (fl)	54.13 ± 1.32	52.33 ± 0.72	52.93 ± 1.51	50.60 ± 0.85	50.76 ± 0.68	51.20 ± 1.12	0.001
MCH (pg)	22.16 ± 0.33	21.16 ± 0.08	21.60 ± 0.79	20.80 ± 0.40	20.90 ± 0.23	21.23 ± 0.38	0.001
MCHC (g/dL)	41.10 ± 0.50	40.53 ± 0.39	40.83 ± 0.33	41.16 ± 0.12	41.33 ± 0.27	41.60 ± 0.15	0.002
PLTs (×10^3^/μL)	630.67 ± 61.48	677.33 ± 4.87	607.00 ± 5.92	667.00 ± 3.81	645.33 ± 3.96	677.33 ± 7.62	<0.001
TLC (10^3^/μL)	4900.0 ± 4.16 ^b^	3866.7 ± 4.97 ^c^	4000.0 ± 7.76 ^b^	4010 ± 7.54 ^b^	4566.7 ± 1.33 ^b^	6700.0 ± 1.85 ^a^	0.001
Neutrophil (%)	12.00 ± 0.57 ^a^	12.66 ± 0.33 ^a^	7.23 ± 4.97 ^c^	7.33 ± 0.66 ^c^	10.33 ± 0.88 ^b^	7.66 ± 1.76 ^c^	0.04
Lymphocyte (%)	83.66 ± 0.66 ^ab^	79.22 ± 3.00 ^c^	88.33 ± 1.30 ^a^	87.00 ± 1.00 ^a^	85.00 ± 1.15 ^ab^	88.00 ± 1.52 ^a^	0.01
Monocyte (%)	2.66 ± 0.33 ^c^	5.00 ± 0.57 ^a^	2.00 ± 0.57 ^c^	3.66 ± 0.66 ^b^	2.66 ± 0.33 ^c^	2.33 ± 0.33 ^c^	<0.001
Eosinophil (%)	1.33 ± 0.33	2.33 ± 0.33	1.66 ± 0.33	1.66 ± 0.33	1.66 ± 0.66	1.66 ± 0.33	0.004
Basophil (%)	0.33 ± 0.33	0.66 ± 0.33	0.33 ± 0.33	0.33 ± 0.33	0.33 ± 0.33	0.33 ± 0.33	0.88
N/L ratio	0.14 ± 0.008 ^b^	0.16 ± 0.01 ^a^	0.08 ± 0.01 ^d^	0.08 ± 0.008 ^d^	0.12 ± 0.01 ^c^	0.09 ± 0.02 ^d^	0.02

Values are represented as means ± SE, *n* = 5. Different superscripted letters (^a, b, c, d^) within the same row indicate statistical significance at *p* < 0.05. TA, thoiacetamide; Sl, silymarin; St, *Spirulina platensis*; Cg, *Chlorella vulgaris*; RBCs, red blood cell counts; Hb, hemoglobin; PCV, packed cell volume; MCV, mean corpuscular volume; MCH, mean corpuscular hemoglobin; MCHC, mean corpuscular hemoglobin concentration; TLC, total leukocytic count; N/L ratio, neutrophils/lymphocyte ratio.

**Table 5:** The valued quantities of cardiac enzymes in the experimental groups.

Cardiac Enzymes	Treatments						
	Control	TA	Sl + TA	St + TA	Cg + TA	St + Cg + TA	*p*-Value
Total CK (IU/L)	34.00 ± 2.91 ^c^	189.00 ± 24.03 ^a^	50.20 ± 11.34 ^c^	111.00 ± 20.24 ^b^	36.20 ± 5.86 ^c^	70.00 ± 13.06 ^bc^	0.03
CK-MB (IU/L)	19.20 ± 3.67 ^c^	62.80 ± 7.78 ^a^	23.40 ± 3.52 ^bc^	36.80 ± 5.86 ^b^	20.60 ± 0.24 ^c^	27.80 ± 2.55 ^bc^	0.01
Troponin I (ng/mL)	0.03 ± 0.003 ^b^	0.13 ± 0.015 ^a^	0.02 ± 0.002 ^b^	0.04 ± 0.003 ^b^	0.03 ± 0.004 ^b^	0.02 ± 0.002 ^b^	0.004

Values are represented as means ± SE, *n* = 5. Different superscripted letters (^a, b, c^) within the same row indicate statistical significance at *p* < 0.05. TA, thoiacetamide; Sl, silymarin; St, *Spirulina platensis*; Cg, *Chlorella vulgaris*. Total CK, total creatine kinase; CK-MB, creatine kinase MB.

**Table 6:** Serum lipid profile of experimental rats.

	Treatments						
Lipid Profile	Control	TA	Sl + TA	St + TA	Cg + TA	St + Cg + TA	*p*-Value
Total lipid (mg/dL)	175.00 ± 11.59	200.00 ± 21.77	164.00 ± 4.82	191.00 ± 16.31	177.00 ± 4.19	174.00 ± 12.57	0.385
Total cholesterol (mg/dL)	36.40 ± 2.20 ^b^	45.60 ± 4.24 ^a^	30.60 ± 1.46 ^c^	36.00 ± 3.39 ^b^	40.00 ± 1.78 ^b^	37.80 ± 3.66 ^b^	0.02
Triglycerides (mg/dL)	91.40 ± 8.05	95.40 ± 12.98	94.40 ± 4.78	108.80 ± 9.08	86.20 ± 7.08	87.80 ± 4.92	0.36
HDL (mg/dL)	11.20 ± 0.80 ^a^	5.80 ± 0.48 ^c^	8.00 ± 0.54 ^b^	8.18 ± 0.90 ^b^	8.76 ± 0.34 ^b^	8.80 ± 1.15 ^b^	0.001
LDL (mg/dL)	11.12 ± 1.22 ^b^	17.82 ± 1.81 ^a^	4.96 ± 1.35 ^c^	6.06 ± 1.27 ^c^	7.72 ± 0.96 ^bc^	11.44 ± 1.85 ^b^	0.03

Values are represented as means ± SE, *n* = 5. Different superscripted letters (^a, b, c^) within the same row indicate statistical significance at *p* < 0.05. TA, thoiacetamide; Sl, silymarin; St, *Spirulina platensis*; Cg, *Chlorella vulgaris*.

**Table 7:** Serum protein parameters.

	Treatments						
Total Proteins	Control	TA	Sl + TA	St + TA	Cg + TA	St + Cg + TA	*p*-Value
Total protein (g/dL)	7.45 ± 0.27 ^b^	6.54 ± 0.29 ^c^	9.23 ± 0.61 ^a^	10.09 ± 1.00 ^a^	7.93 ± 0.10 ^ab^	8.55 ± 0.97 ^a^	0.04
Albumin (g/dL)	3.70 ± 0.18 ^ab^	3.00 ± 0.20 ^c^	3.31 ± 0.08 ^b^	3.75 ± 0.50 ^a^	3.29 ± 0.95 ^b^	3.61 ± 0.03 ^ab^	0.04
Globulin (g/dL)	3.75 ± 0.35 ^bc^	3.54 ± 0.97 ^c^	5.92 ± 0.27 ^a^	6.34 ± 1.08 ^a^	4.64 ± 1.08 ^b^	4.94 ± 0.92 ^b^	0.02
A/G ratio	0.98 ± 0.11 ^a^	0.84 ± 0.48 ^b^	0.56 ± 0.54 ^d^	0.59 ± 0.90 ^d^	0.71 ± 0.34 ^c^	0.73 ± 1.15 ^c^	0.003

Values are represented as means ± SE, *n* = 5. Different superscripted letters (^a, b, c, d^) within the same row indicate statistical significance at *p* < 0.05. TA, thoiacetamide; Sl, silymarin; St, *Spirulina platensis*; Cg, *Chlorella vulgaris*. *A/G* ratio, albumin/globulin ratio.

**Table 8:** Serum oxidant/antioxidant biomarkers.

	Treatments						
Items	Control	TA	Sl + TA	St + TA	Cg + TA	St + Cg + TA	*p*-Value
TAC (mmol/mL)	1.10 ± 0.09 ^a^	0.83 ± 0.27 ^c^	0.95 ± 0.01 ^b^	1.03 ± 0.10 ^b^	1.12 ± 0.11 ^a^	1.30 ± 0.04 ^a^	0.04
MDA (nmol/mL)	5.53 ± 0.06 ^c^	7.56 ± 0.51 ^a^	4.86 ± 0.50 ^c^	6.33 ± 0.25 ^b^	6.46 ± 0.45 ^b^	6.13 ± 0.28 ^b^	0.03

Values are represented as means ± SE, *n* = 5. Different superscripted letters (^a, b, c^) within the same row indicate statistical significance at *p* < 0.05. TA, thoiacetamide; Sl, silymarin; St, *Spirulina platensis*; Cg, *Chlorella vulgaris*. TAC, total antioxidant capacity; MDA, malondialdehyde.

**Table 9:** Histopathological lesion scoring in the heart of experimental groups.

Lesions	Treatments					
	Control	TA	Sl + TA	St + TA	Cg + TA	St + Cg + TA
Vacuolar degeneration of myocytes	0	3	1	2	1	1
Zenker’s necrosis of myocytes	0	3	1	2	1	1
Interstitial fibrosis	0	3	1	2	1	1
Interstitial inflammatory cell infiltration	0	3	1	2	1	0
Interstitial vascular thickening	0	2	0	1	0	0

The score system (1–3) was designed as follows: 0 = absence of the lesion in all rats of the group (*n* = 5), 1 = (<30%), 2 = (<30–50%), 3 = (>50%).

## Data Availability

The data presented in this study are available on request from the corresponding author.
